# Bright Frenkel Excitons in Molecular Crystals: A Survey

**DOI:** 10.1021/acs.chemmater.1c00645

**Published:** 2021-04-23

**Authors:** Tahereh Nematiaram, Daniele Padula, Alessandro Troisi

**Affiliations:** † Department of Chemistry and Materials Innovation Factory, University of Liverpool, Liverpool L69 7ZD, U.K.; ‡ Dipartimento di Biotecnologie, Chimica e Farmacia, Università di Siena, via A. Moro 2, Siena 53100, Italy

## Abstract

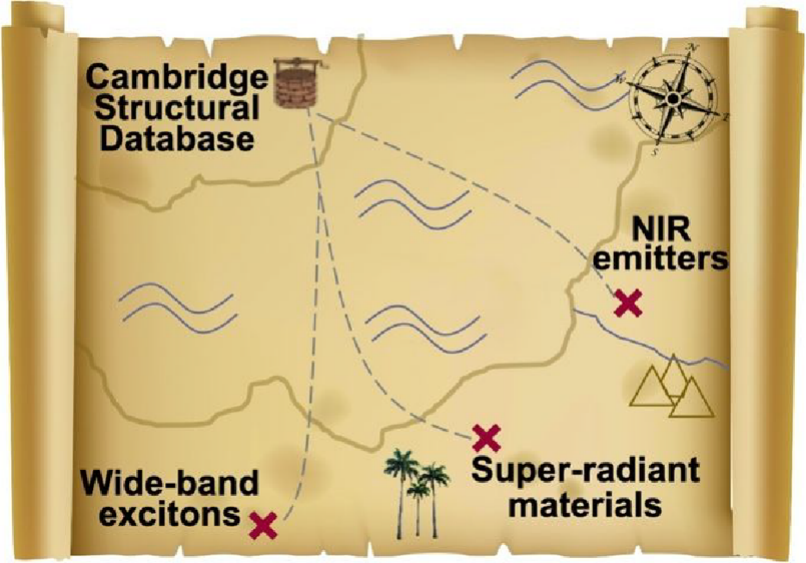

We computed the optical properties of a large set of molecular crystals (∼2200 structures) composed of molecules whose lowest excited states are strongly coupled and generate wide excitonic bands. Such bands are classified in terms of their dimensionality (1-, 2-, and 3-dimensional), the position of the optically allowed state in relation with the excitonic density of states, and the presence of Davydov splitting. The survey confirms that one-dimensional aggregates are rare in molecular crystals highlighting the need to go beyond the simple low-dimensional models. Furthermore, this large set of data is used to search for technologically interesting and less common properties. For instance, we considered the largest excitonic bandwidth that is achievable within known molecular crystals and identified materials with strong super-radiant states. Finally, we explored the possibility that strong excitonic coupling can be used to generate emissive states in the near-infrared region in materials formed by molecules with bright visible absorption and we could identify the maximum allowable red shift in this material class. These insights with the associated searchable database provide practical guidelines for designing materials with interesting optical properties.

## Introduction

Exploring, designing, and synthesis of luminescent molecular crystals, in particular far-red/near-infrared (NIR) emitters (650–1000 nm) and super-radiant structures, exhibiting highly efficient emissions in the solid-state is of great scientific interest.^1−8^ The luminescent molecular crystals find potential application in light-emitting diodes,^9,10^ organic lasers,^11,12^ and biological imaging.^13^ Within this material class, particularly interesting structures are those composed of nonluminescent molecules in solution which turn out to be luminescent in the solid state, a phenomenon initially described by Jelley and Scheibe in the 1930s.^14−15^ An early insightful study with a qualitatively correct explanation of this complex phenomenon was provided in ref.^16^ followed by a full understanding gained a few years later.^8^ The optical properties of molecular crystals are characterized by their numerous electronic and intra- as well as intermolecular excitations.^17−19^ Due to their tunable molecular conformations and packing modes, leading to different intermolecular interactions, these materials provide a rational framework to investigate photophysical properties and explore/design structures that efficiently emit light in the solid state neglecting the complication that might arise in polycrystalline samples or thin films due to their structural defects and grain boundaries.^20−22^ However, crystallinity is often considered as a cause of changes in photochemical properties, for instance, Stokes shift, polarization, and quantum efficiency of fluorescence.^12^ Therefore, to attain high quantum efficiencies suitable for successful technological implementation, the luminescence behavior of these crystalline materials needs to be controlled requiring an understanding of the excited states in the solid-state phase. Accordingly, widespread theoretical and experimental attention has been focused on the mechanism of the electronic excitations in organic solids, predominantly described by Frenkel excitons as a superposition of localized excitations.^23−28^ Throughout the years, researchers have utilized the simple model developed over six decades ago by Kasha and co-workers^29,30^ to understand the photophysics of dimers, as such, molecular dimers stacked in a “side-by-side” fashion result in a positive Coulomb coupling and exhibit a blue-shifted absorption maximum accompanied with a suppressed radiative decay rate and are known as H-aggregates, whereas those packed in a “head-to-tail” configuration exhibit a negative Coulomb coupling leading to a red-shifted absorption maximum and enhanced radiative decay rate and are referred as J-aggregates.^29^ One can shift between H- and J-aggregations through altering the slip or angle between the molecules, and accordingly, many groups have utilized this strategy to generate H- or J-aggregated structures.^31,32^ It is important to note that the difference in the photoluminescence between H- and J-aggregates is related to the radiative decay rates but not to the total emission quantum yield. It is known that the quantum yield depends on how large the radiative rate is compared to the nonradiative rate. Therefore, as shown by Gierschner and co-workers,^18,33^ one can expect highly emissive H-aggregates if the weak radiative decay rate dominates an even lower nonradiative decay rate. The highly emissive H-aggregates are often seen as a result of static and dynamic symmetry breaking, through aggregate-type Herzberg-Teller coupling.^34,35^ Many examples of J- and H- aggregates are experimentally known. The cyanine-based dyes (pseudoisocyanine,^14,36,37^ merocyanine,^38,39^ and thiacarbocyanine^40,41^), perylene diimides,^42,43^ and porphyrins^44,45^ are among the mostly known J-aggregates. H-aggregating chromophores include certain carotenoids,^46,47^ oligothiophenes,^48,49^ oligophenylenevinylenes,^50,51^ as well as perylene diimides,^52,53^ some of which can form both H- and J-aggregation types.^54,55^ Polymer π-stacks such as those based on P3HT are also generally categorized as H-aggregates.^56^

The original Kasha’s theory has been refined in recent years to include the effects of vibronic coupling which were primarily focused on the local electron-vibrational coupling^57−60^ and lately on off-diagonal Peierls coupling, as well.^61−63^ Furthermore, the introduction of the effect of short-range exchange (Dexter^64,65^) and super-exchange interactions have led to new types of aggregates, e.g., the segregated “Hj”-aggregates where the first and second letters indicate the signs of the long- and short-range couplings, respectively, while the upper/lower case is indicative of the relative magnitude of the couplings.^66−68^ Examples of experimentally known segregated aggregates are conjugated polymer π-stacks,^69−71^ crystalline terrylene derivative 7,8,15,16-tetraazaterrylene,^67^ and naphthobisoxadiazole-based copolymer films.^72^ The interested reader is referred to excellent reviews, e.g., refs.^35,73^ for extended discussion on theory generalizations.

Exciton physics in realistic materials is further complicated by the complex network of excitonic couplings (extending to three dimension) which cannot be captured by simple low-dimensional models.^74−82^ As a consequence, despite the considerable progress in the theory generalizations, understanding the characteristic parameters of excitons is yet far from complete. Particularly, the limits of the excitonic bandwidth, an important parameter which has a significant impact on exciton coherent properties, as discussed in experimental and theoretical studies, is still among the interesting questions in the field.^83−88^ Experimental measurements and accurate calculations have shown excitonic bandwidths of as large as ∼0.8–1 eV, e.g., in samples of OPVn, OTn, and para-nitroaniline,^23,89−92^ however, due to the lack of experimental/theoretical studies on a large set of structures, the limit of the exciton bandwidth is not fully understood. Excitonic effects are remarkable when the coupling between excitations localized on different molecules are strong. These strong couplings are mainly due to Coulombic interactions and involve coupling between optically allowed (bright) excited states. Thus, molecular crystals containing molecules with a bright first excited state (S_1_) offer an ideal set of systems to study Frenkel excitons beyond simple low-dimensional models. In this set of systems, the excitonic couplings are larger than the abovementioned interactions (i.e., local electron-vibrational coupling, off-diagonal Peierls coupling, short-range exchange, and super-exchange interactions), and accordingly, optical spectra can be modeled to the first order of approximation due to interacting localized excitations (one per molecule).^93−95^ Most experimentally relevant features can be captured by such a model (e.g., super-radiancy, exciton effective mass, and low energy emission) and other effects can be seen as introducing higher order corrections. Therefore, the aim of the present work is to provide a survey of the optical properties of a large set of known molecular crystals whose lowest excited states are dominated by Frenkel excitons. We will derive a useful classification of materials with strong excitonic character in terms of their bands’ dimensionality, their aggregation type, and the presence of Davydov splitting in their absorption spectra. We will use this classification to discuss the presence of technologically relevant properties within the data set such as super-radiancy and low-energy emissions.

## Methods and Computational Details

### Data Set

Our initial database is a set of 40,000 molecular semiconductors extracted from the Cambridge Structural Database (CSD) for which the excited state energy calculation in their X-ray geometries are performed at the M06-2X/def2-SVP level of theory, as implemented in Gaussian 16,^96^ in a recent work from our group.^97^ In ref.,^97^ a comparison between a set of computed and experimental excitation energies was also used to extract a linear calibration between the two set of data and provide a robust set of molecular excitation energies which are calibrated for spectra in solution and are therefore corrected for the effect of high frequency dielectric screening. This database was recently used to identify novel thermally activated delayed fluorescence materials.^98^ For the purpose of the present study we have reduced the database to a set of materials for which S_1_ is optically allowed and very bright (oscillator strength larger than 0.5), which are expected to generate broad excitonic bands, leading to 2227 crystalline structures.

### Excitonic Hamiltonian

The excitonic Hamiltonian in the absence of couplings to the vibrational modes known as the standard Frenkel exciton model can be expressed as follows in a tight-binding form,^99,100^
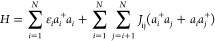
1with ε*_i_* being the excitation energy of the *i*^th^ molecule in the material, *J_ij_* the excitonic coupling between the *i*^th^ and the *j*^th^ molecules, and *a*_*i*_^+^(*a_i_*) the creation (annihilation) operator for an excited state on the *i*^th^ molecule in the material. The excitation energies ε*_i_* can be extracted directly from the TDDFT calculation output. Our analysis reveals that the difference in computed excitation energies of unit cell molecules is negligible (on average ∼0.0034 eV), and accordingly, we use the same excitation energy for all the molecules which will be denoted as ε_0_ hereafter. In these calculations, the coupling with higher excited states is neglected and excitonic couplings *J_ij_* are computed using the transition density cube method.^101^ Using the Multiwfn package,^102^ transition density matrices are discretized into average transition densities for small cubic spatial regions that span the volume encompassing each molecule. The couplings were then calculated as,^101,103^
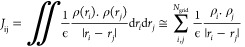
2with ρ(*r_i_*)= ρ*_i_* being the value of the transition density of the localized Frenkel exciton. The dielectric constant ϵ is set to 1,^104^ unless otherwise stated, and while discussing the results, one can keep in mind that the energy bandwidths should be scaled down by the value of this parameter in the bulk crystal.^105^ Within this method, we calculate the excitonic couplings between all nonequivalent pairs of molecules in van der Waals contact,^106^ e.g., molecules such that at least one distance between any two atoms *i* and *j* is shorter than 1.2 × (*r_i_* + *r_j_*) with *r_i_* and *r_j_* being the van der Waals radii from ref.^107^ and also those for which the distance between their mass centers is shorter than 10 Å. Diagonalizing the Hamiltonian enables one to compute the oscillator strength *f_i_* for each transition from the ground to the *i*^th^ excited state of the molecular aggregate as,^108^
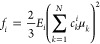
3with μ being the transition dipole moment of the isolated molecule, *N* the total number of molecules, *E_i_* the excitation energies of the aggregate, and *c^i^* the eigenstate expansion coefficients. We consider supercells of size 20 × 20 × 20 (with periodic boundary conditions applied for the supercells) to compute the electronic absorption spectrum as which is normalized by the total number of molecules in the supercell to yield the absorption per molecule. The Hamiltonian parameters from which all the results of this work can be reproduced are given in a searchable format in a GitHub repository.^109^

## Results and Discussion

### Excitonic Bands

**Figure 1:**
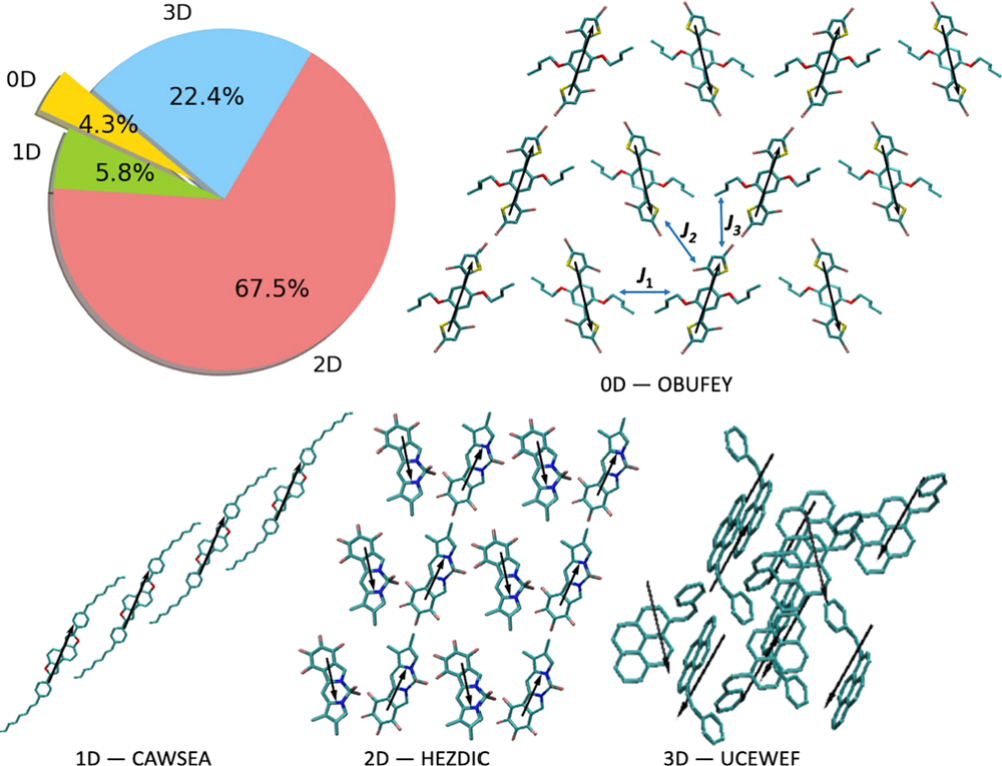
Pie chart representing the percentage of materials with excitonic bands in 0D, 1D, 2D, and 3D alongside representative examples of each category. The structures are labeled with their CSD identifiers and the transition dipole moments are shown by black arrows. The blue arrows indicate the three largest excitonic couplings *J*_1_, *J*_2_, and *J*_3_.

The first analysis of the data was performed to identify the structure sustaining 1-, 2-, and 3D excitonic bands. To this aim and in order to collect the results in an appropriate way, to each excitonic coupling, a vector R is assigned which connects the mass centers of the interacting molecules. The largest and the second largest excitonic couplings in the absolute value whose R vectors are not parallel are denoted as *J*_1_ and *J*_2_. Our results indicate that 4.3% of all materials are those with |*J*_1_| < 0.05 eV, and they are characterized by extremely narrow bands. Since we have selected molecules with bright excited states, this can only happen where the molecules are distant or the transition dipoles are perpendicular to each other. As shown in Figure S1 of the Supporting Information (SI), for about 95% of the cases the dipole–dipole interaction *J*_DD_ is also very small; however, in 5% of the structures, *J*_DD_ is larger than 0.1 eV while *J*_1_ remains below 0.05 eV that is due to the fact that in these structures the higher multipole terms are dominating. We have labeled these structures as 0D excitons as they have very small excitonic bandwidth and retain most of the molecular characteristics. It has to be noted that, for 0D excitons, the main assumption of this work, namely, that the excitonic coupling is stronger than the other couplings of the Hamiltonian, is not valid and this set of structures are excluded from further analysis. To classify the remaining structures in terms of their 1-, 2-, or 3D delocalization, we compute the excitonic band structure considering all the excitonic couplings. If removal of all couplings outside the plane defined by *J*_1_ and *J*_2_ reduces the bandwidth by more than 10%, the excitonic band is defined to be 3D. Among the remaining structures, to differentiate between 1D and 2D materials, we compute the excitonic bands considering all the couplings lying on the plane defined by *J*_1_ and *J*_2_. If removal of the excitonic couplings except *J*_1_ reduces the bandwidth by more than 10%, the excitonic band is defined to be 2D and otherwise is 1D. Accordingly, as shown in the pie chart of Figure 1 (top left), we find that 5.8% of the structures possess 1D excitonic bands, 67.5% have bands in 2D, and 22.4% are 3D. As such, in evaluating photophysical properties, considering the real structure of materials rather than relying on simple low-dimensional models is essential. A few examples of each category represent the adjustment of the molecular pairs and their transition dipole moments.

### Material Aggregation Types

**Figure 2:**
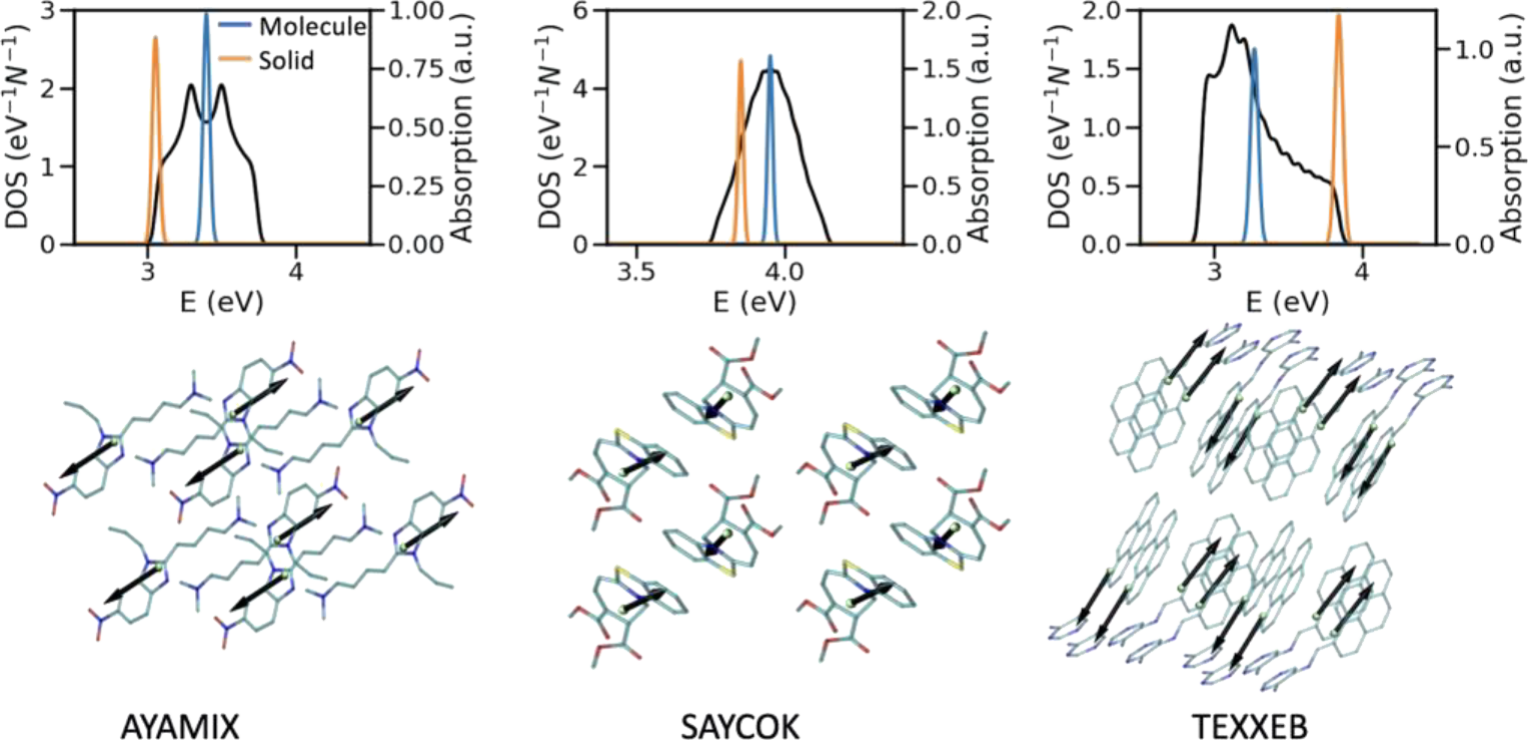
(Top row) Overlapped plot of DOS (shown in black) and the electronic absorption spectra of the single molecule and solid for various aggregation types. Both DOS and absorption are given per molecule. The Gaussian shape function is utilized and the broadening parameter is set to 0.2 ×largest excitonic coupling of the considered structure. The legend shown in the first panel is valid for the other panels as well. The examples are shown among those without Davydov splitting (i.e., possessing only one absorption peak in the electronic absorption spectrum). (Bottom row) Examples of (from left to right) J-, intermediate-, and H-aggregates labeled with their CSD identifiers.

Before providing some statistical analysis on the excitonic state of the database we illustrate some typical examples that will help to define our statistical measures. First, we consider the relation between density of excitonic states (DOS), computed as  and the absorption maximum which, in the simple 1D case and in the absence of vibronic couplings, is associated with pure H- and J-character representing peaks at the top or bottom of the band, respectively. As can be seen in Figure 2, the aggregation type in molecular solids is not limited to the well-known pure H- and J-aggregates (shown in top right and left panels) and there are materials with an “intermediate” aggregation type for which the bright state can be still slightly red(blue)-shifted from the uncoupled molecule but not at the band edges (as in the middle panel).

The second important feature is the presence of Davydov splitting, a common occurrence in structures possessing two or more translationally inequivalent molecules in the unit cell with a relatively strong coupling between the molecules with different transition dipole moments,^110,111^ in many of the considered structures. The commonly studied cases in the literature^23,112,113^ often depict double peaks (DPs) in their absorption spectrum (in the absence of vibronic couplings) while in this work we also identify examples of structures with three peaks (TPs). As such, our analysis shows that 53.1% of the structures, being all 2D and 3D, display Davydov splitting (45% possess two peaks and 8.1% three peaks) and obviously they are not classifiable as pure H- or J-aggregates. There are rare examples of TPs in the literature, e.g., refs.,^114,115^ and it is shown that the positions and the relative intensities of the Davydov peaks depend on the stacking type and the strength of the couplings.^110,116,117^

**Figure 3:**
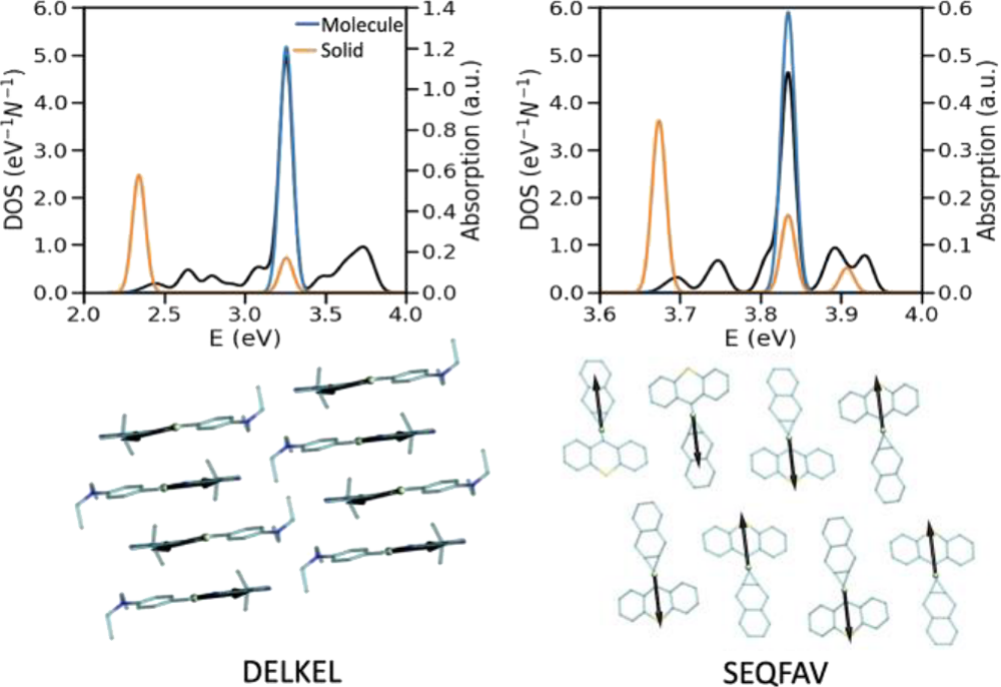
(Top row) Overlapped plot of DOS (shown in black) and the electronic absorption spectra of the single molecule and the solid for examples of materials depicting DPs and TPs in their electronic absorption spectrum. The Gaussian shape function is utilized and the broadening parameter is set to 0.2 ×largest excitonic coupling of the considered structure, and the parameters are given per molecule. (Bottom row) Two representative examples of materials (labeled with their CSD identifiers) depicting DPs and TPs in their electronic absorption spectrum.

Examples of DP and TP absorption spectra alongside the adjustment of the molecular pairs and their transition dipole moments are shown in Figure 3.

The next step is to provide a statistical description of the full set of 1-, 2-, and 3D crystals considering the position of the absorption peak with respect to the DOS and the number of peaks. To this aim, we define a parameter β as the energy difference between the “center” of the absorption in the solid *E*_S_ = ∑ *f_i_E_i_*/ ∑ *f_i_* and the energy of molecule absorption ε_0_ divided by half of the excitonic bandwidth *W*,
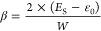
4

**Figure 4:**
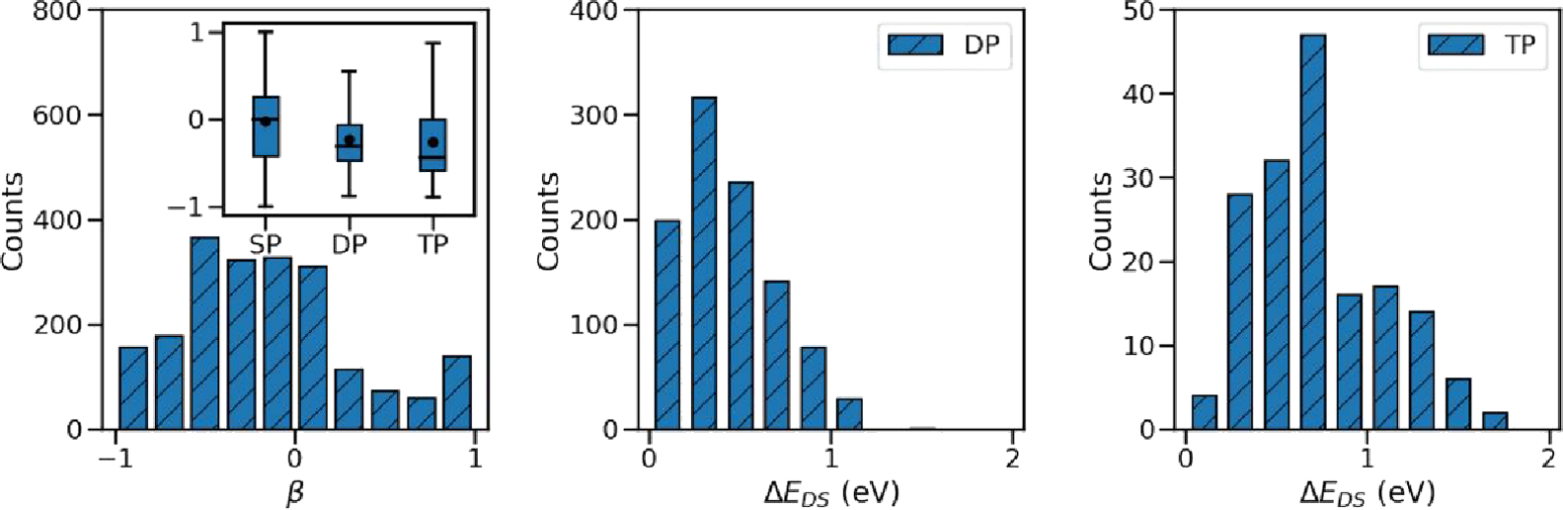
(Left) Distribution of parameter β which indicates the aggregation type. The variation range of β among the materials with single-, double-, and triple-peaks in their electronic absorption spectrum is shown in the inset. The box limits represent the first (Q_1_) and third quartiles (Q_3_), with a line and a small circle representing the median and mean value, respectively. (Middle and Right) Variation range of Δ*E*_DS_ among the materials with double- and triple-peaks in their electronic absorption spectrum.

As such, for a pure H-aggregate, the parameter β is equal to +1 and it is −1 in a pure J-aggregate. The distribution of this parameter shown in Figure 4 (left panel) gives an idea of the number of structures with different aggregation types. As can be seen, 24.1% of the structures are J-like aggregates with β ≤ −0.5, 11.2% are of H-like aggregation types with β ≥ +0.5, and 64.7% are intermediate aggregates and lay outside this classification. In the figure inset, the variation range of β among the structures depicting single-, double-, and triple-peaks in their electronic absorption spectrum is shown in a series of box plots. A comparison between the distribution of the boxes highlights the fact that the materials with Davydov splitting tend to be more of J-like aggregates and the trend becomes more evident in the TP structures (i.e., the median value is closer to −1). It is interesting to note that a well-known class of structures that can be classified within the intermediate aggregates are the so-called segregated HJ-aggregates, described in ref.^35^ These aggregates are two-dimensional structures with one molecule per unit cell where both H- and J-like interactions coexist, leading to a bright state in the band interior. As such, increasing the J(H)-interactions shifts the peak toward the lower (upper) band edge and cancellation between these interactions leads to a bright state at the band center similar to that of the uncoupled molecule.

In the middle and the right panels, the distribution of energy splitting between the Davydov peaks (Δ*E*_DS_) for DP and TP structures are shown. In TP structures, Δ*E*_DS_ is computed as the energy difference between the two peaks which are the farthest. As can be seen, the median and maximum values of Δ*E*_DS_ in TP structures are slightly larger than that of the DP structures. Therefore, in the absence of vibronic couplings, one can anticipate well-separated absorption peaks in the TP structures.

**Figure 5:**
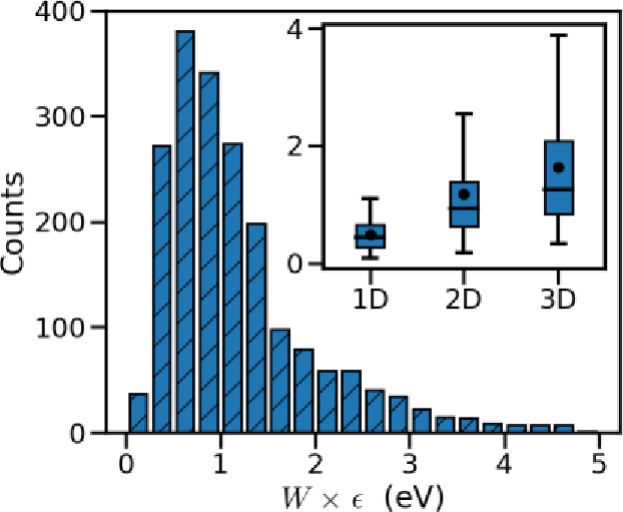
Distribution of the excitonic bandwidth (before scaling by the relative dielectric constant ϵ). The variation range of the bandwidth among the materials with 1-, 2-, and 3D bands is shown in the inset.

The other important parameter that can be explored within this large set of data is the excitonic bandwidth which is shown to strongly affect the exciton delocalization length and relaxation dynamics.^83,88,118,119^ According to the distribution of *W* (before scaling by the dielectric constant ϵ), shown in Figure 5, there are many materials with wide excitonic bandwidths and it is particularly interesting to investigate their common characteristics. First, we note that there is no correlation between *W* and β (rank correlation ∼0.05). The wide bandwidths are more likely found in 2D and 3D excitonic materials, as shown in the inset of Figure 5. There are, however, differences between the 2D and 3D excitonic bands. As such, although the median of *W* in 2D and 3D bands (respectively, 0.94 and 1.25 eV) are relatively close, the larger values of *W* is only seen in 3D bands. Furthermore, our results indicate that in materials with 3D bands, there is a strong correlation of strength +0.64 between the values of the excitonic bandwidth and *J*_3_ (i.e., the largest coupling outside the plane defined by *J*_1_ and *J*_2_). Our results also show that increasing values of the molecular volume and the length of side chains negatively affect the excitonic bandwidths, because of the larger intermolecular distances which lead to smaller excitonic couplings (Supporting Information Figure S2), with correlations of moderate strength −0.29 and −0.35, respectively. Furthermore, we could not find a meaningful correlation with the chemical fingerprints. It is known that the transition dipole moment is a nonlocal property which is only marginally affected by the functional groups and depends mostly on the full topology of the π-conjugated core. The crystal packing is notoriously difficult to predict;^120,121^ therefore, the only chemical conclusion that can be drawn from this study is that bulky functional groups not participating to the lowest energy excitation decrease the importance of all excitonic effects. It is also important to note that the abovementioned criteria for incorporating the neighbors seem adequate because expanding the cut-off to 30 Å for selected crystals, as shown in the Supporting Information (Figure S3), does not change the results significantly.

The presented values of the excitonic bandwidth are obtained with a dielectric constant set to 1. As shown in ref.,^122^ the range of the averaged relative dielectric constant of the molecular semiconductors is mainly in the interval of 2.55–3.34. Considering the mean value of this range (i.e., 2.95) leads to a median bandwidth 0.32 eV and an interquartile range 0.27 eV (Supporting Information Figure S4), which are in excellent agreement with the values reported in refs.^46,123−126^ Furthermore, the attained maximum bandwidth 1.16 eV (considering the top 5% of the data) is in conformity with the theoretical calculations and experimental measurements reported, e.g., in refs.^23,89,127^ and clearly the extreme cases are simply a reflection of the larger data set considered.

It has to be noted that the presented simple scaling by the dielectric constant is only a semiquantitative approach to estimate the effect of dielectric screening and for a more detailed investigation, the distance dependence of ϵ should be also taken into consideration as shown, e.g., in refs.^128−130^ In addition, the effect of dielectric anisotropy is also not considered in our calculations. The range of dielectric anisotropy for different materials is shown to be broad. The mean value of the difference between dielectric constants in perpendicular directions, collected from 12 different experimental reports on 9 different crystals (see Supporting Information Table S1),^131−142^ is Δϵ= 1.32, which can be used as a guide to evaluate the error incurred in assuming the isotropic dielectric response as this work and others, e.g., refs.,^113,143−145^ have done. Studies focusing on a more limited number of cases would benefit from the inclusion of such corrections as shown, e.g., in refs.^146,147^

### Super-Radiant Materials and IR-Emitters

**Figure 6:**
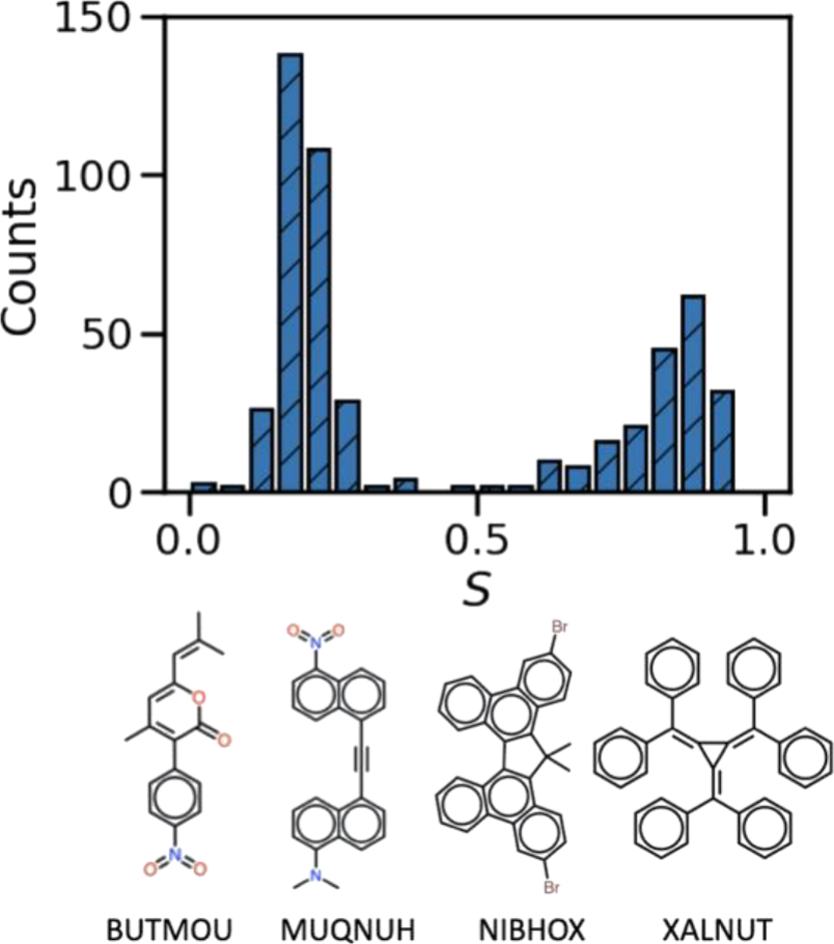
Distribution of super-radiant character *S* and examples of materials with strong super-radiant character.

Super-radiance, the spontaneous radiation emitted from a set of molecules which is faster and stronger than the emission of an independent molecule, is the other important property that this database is screened out for. This phenomenon, which is due to a spontaneous phase-locking of the dipoles,^148,149^ initially was known as a signature of molecular J-aggregates,^150−152^ but then, it was also observed in molecular crystals depicting Davydov splitting such as anthracene^24^ and tetracene,^5,153,154^ highlighting the fact that this property is not limited to pure J-aggregates and can be seen in J-like aggregates as well. Accordingly, we consider all the 512 structures recognized as J-like aggregates in this work to find out the super-radiant structures. We define super-radiant character with *f*_0_ being the oscillator strength of the uncoupled molecule. The weighing by the Boltzmann factor is considered in the *S* definition due to the fact that the emission more likely originates from the thermalized excitonic density of states. Our analysis indicates that the majority of the strong super-radiant structures have multidimensional bands, as such, respectively, 71.1% are 2D, 21.4% possess 3D excitonic bands, and only 7.5% are 1D. This is in good agreement with the observed relation between super-radiancy and the exciton delocalization reported in the literature.^155,156^ The distribution of *S* and representative examples with *S*> 0.85 are given in Figure 6. The full list of super-radiant structures with *S*> 0.5 can be found in the GitHub repository.^109^

**Figure 7:**
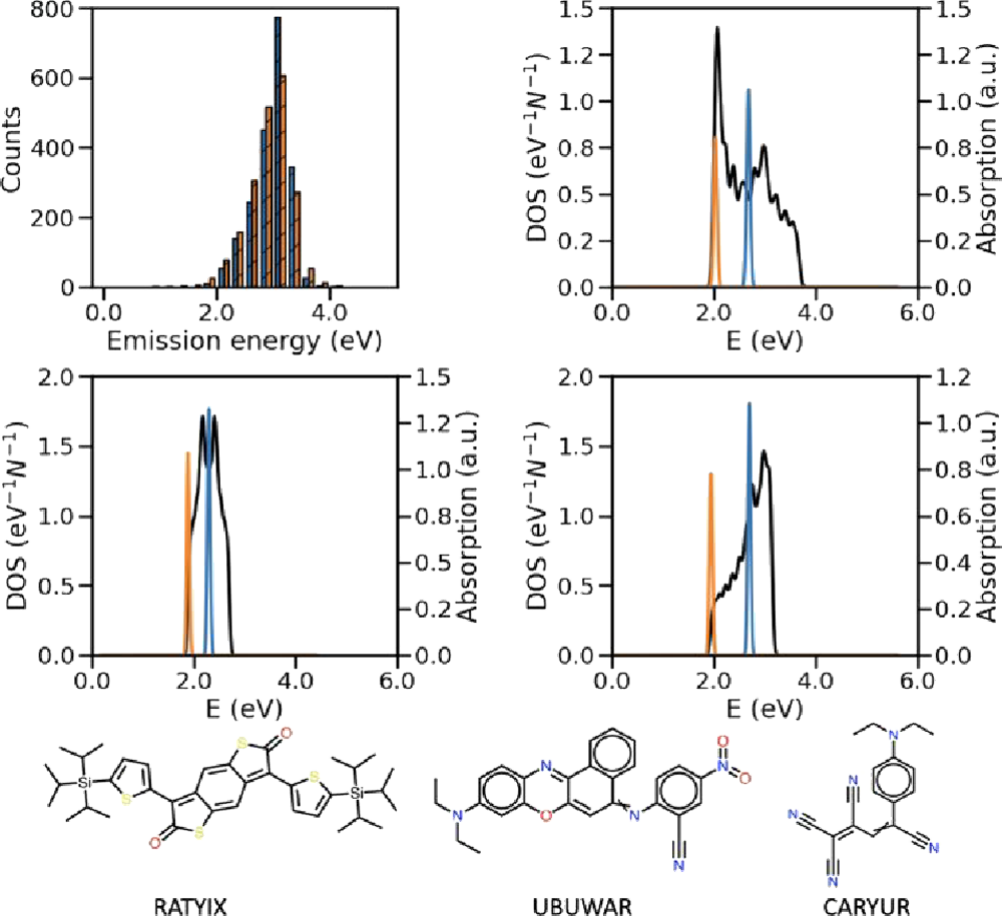
Distribution of emission energy of single molecules and their solid form alongside the electronic absorption spectra of materials emitting at the red edge of the visible spectrum and their molecular diagram labeled with their CSD identifiers.

It is also interesting to search for novel far-red/NIR (with emission energy in the interval 650–1000 nm (1.24–1.91 eV)) emitters in this large set of data. In recent decades, NIR luminescence has become an increasingly popular research field.^157−159^ This has been driven by, on the one hand, the availability of more affordable and sensitive NIR detectors and, on the other hand, the expansion of technological fields where NIR luminescence is an essential tool for analytical detection and transmission of information, for instance, imaging in biological environments,^160^ as well as telecommunications via fiber optics.^161^ Polymethine dyes such as cyanines, pyrrolopyrrole cyanines, squaraines, borondipyrromethenes, and rhodamines are among the experimentally known organic NIR emitters, and expanding the emitter spectrum exploring/designing new compounds is one of the current objectives in the field.^162−164^ At this stage, to make predictions more accurate, we also consider the impact of the dielectric constant by setting it to the above explained averaged value 2.95. As stated above, the environment effect is not explicitly considered in molecular excitation energy calculations. However, since the optical dielectric constants of solvents and that of solids are similar, using the calibrated values of S_1_ energies to reproduce the absorption in solution (as we have done here) will at the same time correct inaccuracies in the computed excitation energy and take into account the effect of the dielectric environment. The results indicate that, as a consequence of forming molecular aggregates, the emission energy of three molecular crystals falls on the red edge of the visible spectrum (i.e., emission energy smaller than 2.10 eV) but not yet in the NIR spectral range. These three structures (being all 2D and 3D) are of J-like aggregates and as our results show, their super-radiancy character lies in the interval of top 30–50% values of *S*. The emission energies of the single molecule and the solid alongside the electronic absorption spectrum of special interesting cases of super-radiant materials, i.e., all these three structures emitting at low energies, with their molecular structures are given in Figure 7. The distribution of the difference in molecular and solid emission energies is represented in the Supporting Information (Figure S5) showing a median value 0.11 eV with a maximum, considering the top 5%, 0.56 eV (with the extreme maximum being 1.42 eV). As such, the results are in accordance with the majority of values reported in the literature, e.g., refs.^6,31,165−167^ Therefore, according to these results, one can expect to achieve a red shift of ∼0.6 eV in properly engineered molecular solids with strong excitonic characters and such shift can be used to design low-energy emitters starting with molecules with low excitation energy in solution.

For verification of the findings of this work against experimental measurements, a comparison with experimental spectra is provided in Figure S6 (Supporting Information). However, as our model does not include vibronic coupling, the comparison is not sufficiently stringent to validate our results. We have therefore compared the excitonic coupling computed in this work with those extracted from other theoretical work that, by including vibronic coupling, have successfully reproduced experimental spectra or other observables. We have reported such comparison in Table S2 (Supporting Information) and the results are fully satisfactory. This observation, alongside the already satisfactory conditions concerning the bandwidth and the extent of red shift, confirm that the results presented in this work are of acceptable precision and the identified materials with intriguing optical properties constitute a robust set of structures to be considered for experimental evaluations.

## Conclusions

We evaluated the optical properties of a large set of known molecular crystals (extracted from the Cambridge Structural Database) with their main feature being to possess optically allowed S_1_ with a large oscillator strength. We classified these structures in terms of their bands’ dimensionality, the position of their absorption peak with respect to the excitonic DOS, and the number of peaks. Our results indicated that the formation of one-dimensional aggregates is a rare occurrence in molecular crystals, and therefore, in evaluating photophysical properties, it is essential to consider the real structure of a material rather than relying on simple low dimensional models. In addition, the wider bandwidths are mainly found in materials with multidimensional excitonic bands particularly in those with smaller intermolecular distances. We also provided a detailed analysis of materials with Davydov splitting observing double- and also triple-peaks in the electronic absorption spectra of structures possessing numerous translationally inequivalent unit cell molecules where, in the absence of vibronic couplings, TP structures presented well-separated absorption peaks. The super-radiancy and low-energy emissions, as interesting yet less evaluated technologically relevant properties, were also searched in this database. As such, we could identify a large set of super-radiant materials displaying diverse structures that could be potentially exploited not only in respective optoelectronic applications but also to initiate new lines of investigations. While the screening did not reveal any structure emitting sharply at the NIR spectral region, the observed possible large energy difference between the molecular and solid emission energies is promising as it indicates that the maximum allowable red shift can be developed in properly engineered materials with strong excitonic character. We believe these insights with the associated searchable database^109^ provide a very broad overview of this class of materials offering practical guidelines for designing materials with useful optical properties.

## Supporting Information Available

Excitonic coupling versus dipole–dipole interaction for 0D structures; excitonic coupling versus intermolecular distances; excitonic bandwidth with an expanded cut-off 30 Å; excitonic bandwidth scaled by the dielectric constant; impact of dielectric anisotropy on the excitonic bandwidth; distribution of difference between molecules’ and solids’ emission energies; and comparison with experimental measurements (PDF)
