# Supplementary material for: Bright Frenkel Excitons in Molecular Crystals: A Survey
Source: Chem Mater. 2021 Apr 23;33(9):3368–78. doi: 10.1021/acs.chemmater.1c00645 (PMC8432684; doi:10.1021/acs.chemmater.1c00645)
Supplement: Supplementary file 1 — cm1c00645_si_001.pdf [file cm1c00645_si_001.pdf]

SUPPORTING INFORMATION FOR:

Bright Frenkel excitons in molecular crystals: a survey

Tahereh Nematiaram,[1*] Daniele Padula,[2] and Alessandro Troisi[1*]
[1]Dept. of Chemistry and Materials Innovation Factory, University of Liverpool, Liverpool L69 7ZD, U.K.
[2]Dipartimento di Biotecnologie, Chimica e Farmacia, Università di Siena, via A. Moro 2, 53100, Siena, Italy
tahereh.nematiaram@liverpool.ac.uk
a.troisi@liverpool.ac.uk

Contents

A. Excitonic coupling versus dipole-dipole interaction for 0D structures

B. Excitonic coupling versus intermolecular distances

C. Excitonic bandwidth with an expanded cut-off 30 Å

D. Excitonic bandwidth scaled by dielectric constant

E. Impact of dielectric anisotropy on the excitonic bandwidth

F. Distribution of difference between molecules' and solids' emission energies

G. Comparison with experimental measurements

A. Excitonic coupling versus dipole-dipole interaction for 0D structures

Our results indicated that 4.3% of all materials have $|J_1| < 0.05$ eV and they are characterized by extremely narrow bands. Since we have selected molecules with bright excited states, this can only happen where the molecules are distant or the dipoles are perpendicular to each other. As shown in Figure S1 (left panel), for about 95% of the cases the dipole-dipole interaction J_{DD} is also very small, however, in 5% of the structures J_{DD} is larger than 0.1 eV while J_1 remains below 0.05 eV that is due to the fact that in these structures the higher multipole terms are dominating. We have labelled these structures as 0D excitons as they have very small excitonic bandwidth and retain most of the molecular characteristics and are excluded from further analysis. The molecular diagram of materials depicting larger dipole-dipole interactions are shown in the right panel.

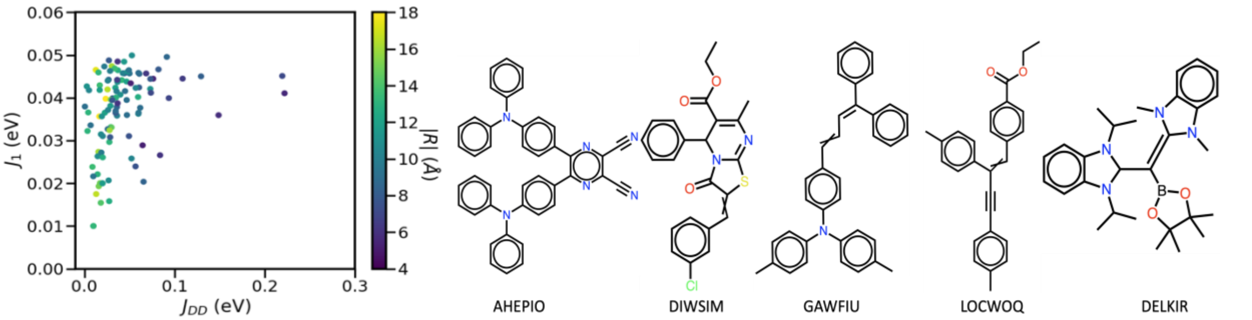

Figure S1. (Left) A scatter plot showing the relation between the computed excitonic coupling and the dipole-dipole interaction. The points in the plot are colored based on the distance of their mass centers. (Right) The molecular diagram of structures depicting significantly larger dipole-dipole interactions.

B. Excitonic coupling versus intermolecular distances

As stated in the main manuscript, our results indicate that increasing values of molecular volume and the length of side chains negatively affect excitonic band widths, because of the larger intermolecular distances. In Figure S2, the relation between the largest excitonic coupling and the associated intermolecular distance is shown. As can be seen, there is a relatively large rank correlation of strength −0.65 between the two parameters reflecting the fact that the closely packed molecular pairs are expected to yield larger values of excitonic couplings.

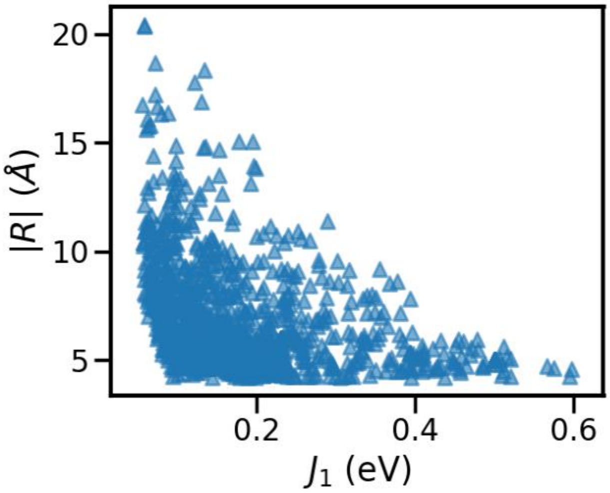

Figure S2. A scatter plot reflecting the relation between the computed excitonic coupling and the associated intermolecular distance.

C. Excitonic bandwidth with an expanded cut-off 30 Å

As explained in the main manuscript, in these calculations, we have considered all molecular pairs in van der Waals contact and also those with mass centers closer than 10 Å. As was shown in Figure S2, with the considered criteria, pairs distanced up to 20 Å are included in our calculations. This implies that the majority of “important” excitonic couplings have been considered within the defined cut-off. However, to investigate this in further detail, we have computed the excitonic bandwidth of 100 structures with a relaxed cut-off 30 Å finding a correlation with strength +0.97 (Figure S3) between the two set of computed bandwidths W_1 (with cut-off 10 Å) and W_2 (with cut-off 30 Å) confirming the validity of the initially defined threshold.

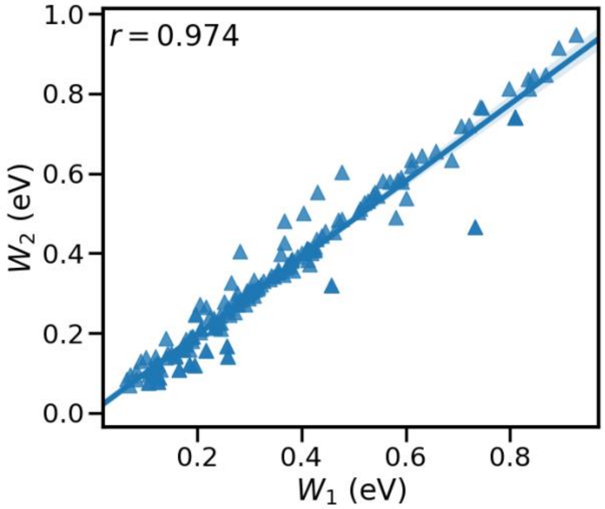

Figure S3. The regression plot between the computed bandwidths W_1 (with cut-off 10 Å) and W_2 (with cut-off 30 Å).

D. Excitonic bandwidth scaled by dielectric constant

In the main manuscript, the excitonic bandwidths are obtained with a dielectric constant set to 1. As shown in Figure S4, considering an averaged dielectric constant 2.95, leads to median bandwidth 0.32 eV and an interquartile range 0.27 eV which are in excellent agreement with the values reported in Refs.[1–5]. Furthermore, the attained maximum bandwidth 1.16 eV (considering the top 5% of the data) is in conformity with the values reported e.g. in Refs.[6–8].

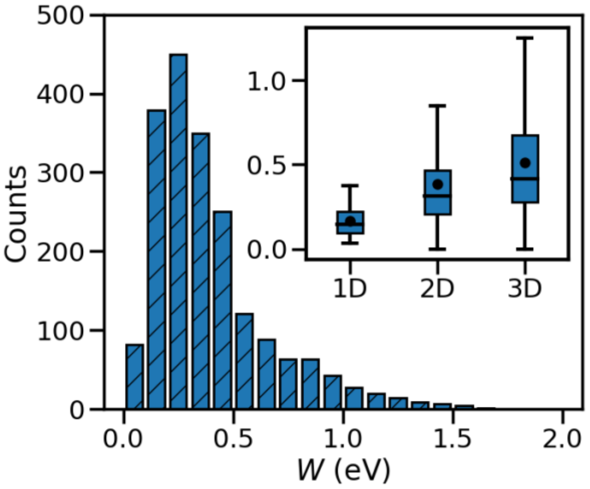

Figure S4. The distribution of excitonic bandwidth scaled by an averaged dielectric constant 2.95. The variation range of W among the materials with 1-, 2-, and 3D bands is shown in the inset.

E. Impact of dielectric anisotropy on the excitonic bandwidth

It is clear that when performing a large-scale screening, one cannot afford the use of the most accurate possible methods. A survey of current literature reveals that the majority of current studies do not consider the impact of dielectric constant, e.g. Refs.[9–11], where the number of considered crystals are significantly fewer than the one studied in this work. On the other hand, the range of dielectric anisotropy for different materials is shown to be broad. It was instructive to collect this information in Table S1, where the range of dielectric anisotropy reported in the literature is given. It has to be noted that the refractive index when measured with frequency that is absorbed by the sample develops a special character which depends on the exciton and that cannot be precisely counted as anisotropy. Accordingly, the values reported in this table are for non-resonant frequencies. The mean value of the difference between dielectric constant in perpendicular directions, collected from 12 different experimental reports on 9 different crystals, is $\Delta\epsilon = 1.32$ which can be used as a guide to evaluate the error incurred in assuming isotropic dielectric response as this work and others, e.g. Refs.[12–15], have done. Studies focusing on a more limited number of cases would benefit from the inclusion of such corrections as shown e.g. in Refs.[16,17].

Table S1. The values of dielectric anisotropy reported in the literature.

Molecule	Reference	Frequency	Values in the paper	Dielectric anisotropy
PTCDA	[18]	9406 cm^{-1}	$n_{\perp} = 1.36 \pm 0.01$<br>$n_{\parallel} = 2.017 \pm 0.005$<br>$\Delta n = 0.66$<br>$\epsilon_{\perp} = 1.9 \pm 0.1$<br>$\epsilon_{\parallel} = 4.5 \pm 0.2$	$\Delta\epsilon_{Max} = 2.90$
PTCDA	[19]	independent of frequency	$n_{\alpha} = 1.55$<br>$n_{\beta} = 2.30$<br>$n_{\gamma} = 2.24$	$\Delta\epsilon_{Max} = 2.88$
Anthracene	[20]	independent of frequency	$\epsilon_{11} = 2.51$<br>$\epsilon_{22} = 2.99$<br>$\epsilon_{33} = 4.11$	$\Delta\epsilon_{Max} = 1.6$
Anthracene	[21]	independent of frequency	$\epsilon_{11} = 2.62$<br>$\epsilon_{22} = 2.94$<br>$\epsilon_{33} = 4.08$	$\Delta\epsilon_{Max} = 1.46$
naphthalene	[22]	53103 cm^{-1}	$\epsilon_{11} = 2.25 \pm 0.07$<br>$\epsilon_{22} = 2.87 \pm 0.05$<br>$\epsilon_{33} = 3.43 \pm 0.08$	$\Delta\epsilon_{Max} = 1.18$
Rubrene	[23,24]	33876 cm^{-1}	$n_{\alpha} = 1.4$<br>$n_{\beta} = 1.5$<br>$n_{\gamma} = 1.7$<br>(extracted from Figure 2c/ Figure 4 of second ref.)	$\Delta\epsilon_{Max} = 0.93$
Pentacene	[25]	20164 cm^{-1}	$\epsilon_{11} = 2.9$<br>$\epsilon_{22} = 4.2$<br>$\epsilon_{33} = 2.9$<br>(extracted from Figure 3)	$\Delta\epsilon_{Max} = 1.3$
Pyrene	[26]	201638 cm^{-1}	$\epsilon_{11} = 0.6$<br>$\epsilon_{22} = 0.4$<br>$\epsilon_{33} = 0.2$<br>(extracted from Figure 2a-c)	$\Delta\epsilon_{Max} = 0.4$
quaterthiophene	[27]	48300 cm^{-1}	$\epsilon_{11} = 1.7$<br>$\epsilon_{22} = 2.3$<br>$\epsilon_{33} = 1.1$<br>(extracted from Figure 1)	$\Delta\epsilon_{Max} = 1.2$
Tetracene	[28]	25800 cm^{-1}	$n_{\perp} = 1.5$<br>$n_{\parallel} = 1.43$<br>(extracted from Figure 5)	$\Delta\epsilon_{Max} = 0.21$
Oligothiophene	[29]	40000 cm^{-1}	$n_{\perp} = 1.4$<br>$n_{\parallel} = 1.2$<br>(extracted from Figure 5)	$\Delta\epsilon_{Max} = 0.52$

F. Distribution of difference between molecules' and solids' emission energies

The distribution of difference in molecular and solid emission energies (in absolute value) is represented in Figure S5 showing a median value 0.11 eV and a maximum (considering the top 5%) 0.56 eV (with extreme maximum being 1.42 eV) which is in accordance with majority of values reported in the literature, e.g. Refs.[30–34].

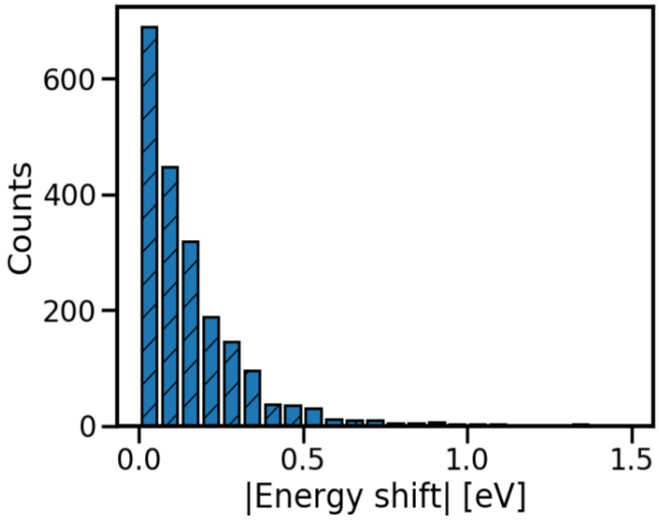

Figure S5. The distribution of energy difference between molecular and solid emission energies in absolute value.

G. Comparison with experimental measurements

A comparison with experimental spectra is provided in Figure S6 for Pentacene and Rubrene crystals (isotropic absorption). Computed absorption spectra are scaled in intensity and shifted in energy by −0.20 and −0.28 eV. The computed spectra are consistent with the experiment (the energy splitting between the electronic peaks is slightly underestimated with respect to the experiment) but, overall, this comparison is not sufficiently stringent to validate our results because we have not included vibronic effects. We have therefore compared the excitonic coupling computed in this work with those extracted from other theoretical work that by including vibronic coupling have successfully reproduced experimental spectra or other observables. We report such comparison in Table S2 and the results are fully satisfactory.

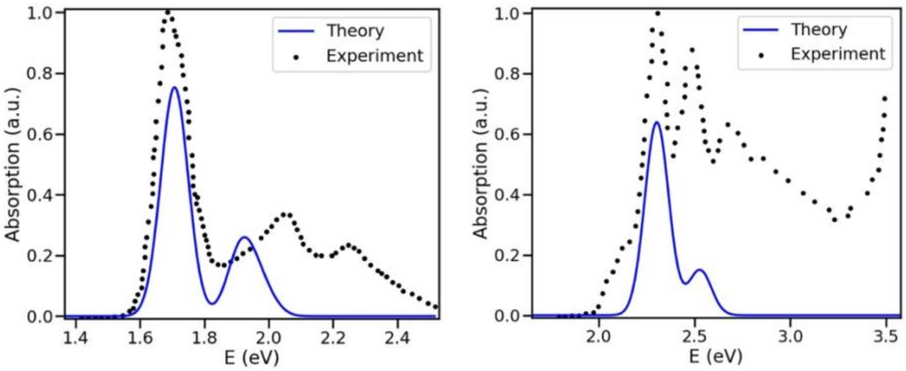

Figure S6. Calculated absorption spectra along with experiment spectra for (left) Pentacene and (right) Rubrene. Experimental and the calculated spectra are normalized and aligned by an energy shift of −0.2 eV and −0.28 eV in the left and right panels, respectively. For Pentacene the experimental spectrum is digitalized from Ref.[35] which is based on analysis of Ref.[36]. The dataset used for experimental spectrum of Rubrene is based on Ref.[37].

Table S2. The values of excitonic couplings computed in this work compared and also those reported in literature.

**Molecule (CCDC ID)**	***J* (meV)**	***J* (meV) – other works**
IZEPIM01	114.86	131 Ref.[38]
GOZHOS	89.97	103 Ref.[38]
REDHIR	104.23	97 Ref.[38]
ANUYEO	44.70	53 Ref.[38]
DEXQEF	101.03	113 Ref.[38]
TETCEN	44.12	41.85 Ref.[39]
PENCEN	32.8	27.4 Ref.[13]

References

(1) Clark, J.; Chang, J.-F.; Spano, F. C.; Friend, R. H.; Silva, C. Determining Exciton Bandwidth and Film Microstructure in Polythiophene Films Using Linear Absorption Spectroscopy. *Appl. Phys. Lett.* **2009**, *94* (16), 163306.

(2) Totoki, R.; Aoki-Matsumoto, T.; Mizuno, K. Density of States of the Lowest Exciton Band and the Exciton Bandwidth in Coronene Single Crystals. *J. Lumin.* **2005**, *112* (1–4), 308–311.

(3) Tempelaar, R.; Stradomska, A.; Knoester, J.; Spano, F. C. Circularly Polarized Luminescence as a Probe for Long-Range Interactions in Molecular Aggregates. *J. Phys. Chem. B* **2011**, *115* (36), 10592–10603.

(4) Drobizhev, M.; Sigel, C.; Rebane, A. Picosecond Fluorescence Decay and Exciton Dynamics in a New Far-Red Molecular J-Aggregate System. *J. Lumin.* **2000**, *86* (2), 107–116.

(5) Spano, F. C. Analysis of the UV/Vis and CD Spectral Line Shapes of Carotenoid Assemblies: Spectral Signatures of Chiral H -Aggregates. *J. Am. Chem. Soc.* **2009**, *131* (12), 4267–4278.

(6) Egelhaaf, H. J.; Bäuerle, P.; Rauer, K.; Hoffmann, V.; Oelkrug, D. Orientation and Mobility in Ultrathin Oligothiophene Films: UV-Vis, IR and Fluorescence Studies. *Synth. Met.* **1993**, *61* (1–2), 143–146.

(7) Spano, F. C. Excitons in Conjugated Oligomer Aggregates, Films, and Crystals. *Annu. Rev. Phys. Chem.* **2006**, *57* (1), 217–243.

(8) Liu, Y.; Guo, Y.; Liu, Y. High-Mobility Organic Light-Emitting Semiconductors and Its Optoelectronic Devices. *Small Struct.* **2021**, *2* (1), 2000083.

(9) Spano, F. C. The Spectral Signatures of Frenkel Polarons in H- and J-Aggregates. *Acc. Chem. Res.* **2010**, *43* (3), 429–439.

(10) Linares, M.; Beljonne, D.; Cornil, J.; Lancaster, K.; Brédas, J.-L.; Verlaak, S.; Mityashin, A.; Heremans, P.; Fuchs, A.; Lennartz, C.; et al. On the Interface Dipole at the Pentacene−Fullerene Heterojunction: A Theoretical Study. *J. Phys. Chem. C* **2010**, *114* (7), 3215–3224.

(11) Ahn, T.-S.; Müller, A. M.; Al-Kaysi, R. O.; Spano, F. C.; Norton, J. E.; Beljonne, D.; Brédas, J.-L.; Bardeen, C. J. Experimental and Theoretical Study of Temperature Dependent Exciton Delocalization and Relaxation in Anthracene Thin Films. *J. Chem. Phys.* **2008**, *128* (5), 054505.

(12) Scholz, R.; Vragovic, I.; Kobitski, A. Y.; Schreiber, M.; Wagner, H. P.; Zahn, D. R. T. Frenkel Exciton Model of Low Temperature Photoluminescence in Alpha-PTCDA Single Crystals. *Phys. status solidi* **2002**, *234* (1), 402–410.

(13) Hestand, N. J.; Yamagata, H.; Xu, B.; Sun, D.; Zhong, Y.; Harutyunyan, A. R.; Chen, G.; Dai, H.-L.; Rao, Y.; Spano, F. C. Polarized Absorption in Crystalline Pentacene: Theory vs Experiment. *J. Phys. Chem. C* **2015**, *119* (38), 22137–22147.

(14) Hestand, N. J.; Spano, F. C. Interference between Coulombic and CT-Mediated Couplings in Molecular Aggregates: H- to J-Aggregate Transformation in Perylene-Based π-Stacks. *J. Chem. Phys.* **2015**, *143* (24), 244707.

(15) Kocherzhenko, A. A.; Sosa Vazquez, X. A.; Milanese, J. M.; Isborn, C. M. Absorption Spectra for Disordered Aggregates of Chromophores Using the Exciton Model. *J. Chem. Theory Comput.* **2017**, *13* (8), 3787–3801.

(16) Sai, N.; Tiago, M. L.; Chelikowsky, J. R.; Reboredo, F. A. Optical Spectra and Exchange-Correlation Effects in Molecular Crystals. *Phys. Rev. B* **2008**, *77* (16), 161306.

(17) Andrzejak, M.; Petelenz, P. Polarization Energy Calculations of Charge Transfer States in the α-Sexithiophene Crystal. *Synth. Met.* **2000**, *109* (1–3), 97–100.

(18) Zang, D. Y.; So, F. F.; Forrest, S. R. Giant Anisotropies in the Dielectric Properties of Quasi-epitaxial Crystalline Organic Semiconductor Thin Films. *Appl. Phys. Lett.* **1991**, *59* (7), 823–825.

(19) Alonso, M. I.; Garriga, M.; Karl, N.; Ossó, J. O.; Schreiber, F. Anisotropic Optical Properties of Single Crystalline PTCDA Studied by Spectroscopic Ellipsometry. *Org. Electron.* **2002**, *3* (1), 23–31.

(20) Karl, N.; Rohrbacher, H.; Siebert, D. Dielectric Tensor and Relaxation of Photoexcited Charge Carriers in Single Crystal Anthracene in an Alternating Field without Direct Contacts. *Phys. Status Solidi* **1971**, *4* (1), 105–109.

(21) Munn, R. W.; Nicholson, J. R.; Schwob, H. P.; Williams, D. F. Dielectric Tensor of Anthracene as a Function of Temperature and Pressure. *J. Chem. Phys.* **1973**, *58* (9), 3828–3832.

(22) Munn, R. W.; Williams, D. F. Dielectric Tensor and the Local Electric Field in Naphthalene. *J. Chem. Phys.* **1973**, *59* (4), 1742–1746.

(23) Tavazzi, S.; Borghesi, A.; Papagni, A.; Spearman, P.; Silvestri, L.; Yassar, A.; Camposeo, A.; Polo, M.; Pisignano, D. Optical Response and Emission Waveguiding in Rubrene Crystals. *Phys. Rev. B* **2007**, *75* (24), 245416.

(24) Tavazzi, S.; Silvestri, L.; Campione, M.; Borghesi, A.; Papagni, A.; Spearman, P.; Yassar, A.; Camposeo, A.; Pisignano, D. Generalized Ellipsometry and Dielectric Tensor of Rubrene Single Crystals. *J. Appl. Phys.* **2007**, *102* (2), 023107.

(25) Dressel, M.; Gompf, B.; Faltermeier, D.; Tripathi, A. K.; Pflaum, J.; Schubert, M. Kramers-Kronig-Consistent Optical Functions of Anisotropic Crystals: Generalized Spectroscopic Ellipsometry on Pentacene. *Opt. Express* **2008**, *16* (24), 19770.

(26) Venghaus, H. Determination of the Dielectric Tensor of Pyrene (C16H10) by Means of Electron Energy Loss Measurements. *Phys. Status Solidi* **1972**, *54* (2), 671–679.

(27) Tavazzi, S.; Borghesi, A.; Campione, M.; Laicini, M.; Trabattoni, S.; Spearman, P. Reflectivity and Anisotropic Optical Functions of Quaterthiophene Single Crystals. *J. Chem. Phys.* **2004**, *120* (15), 7136–7140.

(28) Tavazzi, S.; Raimondo, L.; Silvestri, L.; Spearman, P.; Camposeo, A.; Polo, M.; Pisignano, D. Dielectric Tensor of Tetracene Single Crystals: The Effect of Anisotropy on Polarized Absorption and Emission Spectra. *J. Chem. Phys.* **2008**, *128* (15), 154709.

(29) Egelhaaf, H.-J.; Gierschner, J.; Haiber, J.; Oelkrug, D. Optical Constants of Highly Oriented Oligothiophene Films and Nanoparticles. *Opt. Mater. (Amst).* **1999**, *12* (2–3), 395–401.

(30) Bricks, J. L.; Slominskii, Y. L.; Panas, I. D.; Demchenko, A. P. Fluorescent J-Aggregates of Cyanine Dyes: Basic Research and Applications Review. *Methods Appl. Fluoresc.* **2017**, *6* (1), 012001.

(31) Würthner, F.; Kaiser, T. E.; Saha-Möller, C. R. J-Aggregates: From Serendipitous Discovery to Supramolecular Engineering of Functional Dye Materials. *Angew. Chemie Int. Ed.* **2011**, *50* (15), 3376–3410.

(32) Deshmukh, A. P.; Koppel, D.; Chuang, C.; Cadena, D. M.; Cao, J.; Caram, J. R. Design Principles for Two-Dimensional Molecular Aggregates Using Kasha's Model: Tunable Photophysics in Near and Short-Wave Infrared. *J. Phys. Chem. C* **2019**, *123* (30), 18702–18710.

(33) Guerrini, M.; Calzolari, A.; Corni, S. Solid-State Effects on the Optical Excitation of Push–Pull Molecular J-Aggregates by First-Principles Simulations. *ACS Omega* **2018**, *3* (9), 10481–10486.

(34) Würthner, F.; Saha-Möller, C. R.; Fimmel, B.; Ogi, S.; Leowanawat, P.; Schmidt, D. Perylene Bisimide Dye Assemblies as Archetype Functional Supramolecular Materials. *Chem. Rev.* **2016**, *116* (3), 962–1052.

(35) Beljonne, D.; Yamagata, H.; Brédas, J. L.; Spano, F. C.; Olivier, Y. Charge-Transfer Excitations Steer the Davydov Splitting and Mediate Singlet Exciton Fission in Pentacene. *Phys. Rev. Lett.* **2013**, *110* (22), 226402.

(36) Sebastian, L.; Weiser, G.; Bässler, H. Charge Transfer Transitions in Solid Tetracene and Pentacene Studied by Electroabsorption. *Chem. Phys.* **1981**, *61* (1–2), 125–135.

(37) Ma, L.; Zhang, K.; Kloc, C.; Sun, H.; Michel-Beyerle, M. E.; Gurzadyan, G. G. Singlet Fission in Rubrene Single Crystal: Direct Observation by Femtosecond Pump–Probe Spectroscopy. *Phys. Chem. Chem. Phys.* **2012**, *14* (23), 8307.

(38) Rivera, M.; Stojanović, L.; Crespo-Otero, R. Role of Conical Intersections on the Efficiency of Fluorescent Organic Molecular Crystals. *J. Phys. Chem. A* **2021**, acs.jpca.0c11072.

(39) Xie, X.; Ma, H. Opposite Anisotropy Effects of Singlet and Triplet Exciton Diffusion in Tetracene Crystal. *ChemistryOpen* **2016**, *5* (3), 201–205.
